# Spinal Cord Infarction in a Young Patient After a Simple Stretching Maneuver: A Case Report of Rare Etiology and Diagnostic Challenges

**DOI:** 10.7759/cureus.112620

**Published:** 2026-07-13

**Authors:** Normalizah Abd Manap, Mahedzan Mat Rabi, Rafidah Zainon

**Affiliations:** 1 Department of Radiology, School of Medical Sciences, Universiti Sains Malaysia, Kota Bharu, MYS; 2 Department of Radiology, Hospital Seberang Jaya, Seberang Perai, MYS; 3 Department of Biomedical Imaging, Pusat Kanser Tun Abdullah Ahmad Badawi, Universiti Sains Malaysia, Kepala Batas, MYS

**Keywords:** cervical spine, minor trauma, neuroimaging, spinal cord infarction, young patient

## Abstract

Spinal cord infarction is a rare and diagnostically challenging condition, particularly in young patients without significant trauma or identifiable vascular risk factors. It typically presents with acute motor deficits, impaired pain and temperature sensation, and relative preservation of proprioception. We report a case of a 17-year-old male who developed sudden bilateral upper limb weakness and numbness following a simple stretching maneuver while studying, during which an audible “pop” was noted. His symptoms rapidly progressed to involve the lower limbs, resulting in an inability to ambulate. Magnetic resonance imaging (MRI) of the cervical spine, performed approximately 24 hours after symptom onset, demonstrated longitudinally high T2-weighted signal intensity extending from C5 to T3, with predominant anterior column involvement, consistent with spinal cord infarction. Extensive serological investigations excluded alternative diagnoses, including inflammatory and demyelinating conditions such as transverse myelitis. This case highlights the importance of early imaging and a high index of clinical suspicion when evaluating acute neurological deficits in young patients with atypical presentations.

## Introduction

Spinal cord infarction (SCI) is a rare but potentially devastating neurological condition, accounting for approximately 1-2% of all strokes [1]. It is most commonly associated with identifiable vascular risk factors such as atherosclerosis, hypotension, aortic pathology, or embolic events [2]. The anterior spinal artery supplies the anterior two-thirds of the spinal cord. Ischemia in this area usually causes anterior spinal cord syndrome, which is marked by sudden motor problems, loss of pain and temperature sensation, and relative preservation of proprioception [3].

However, SCI in young individuals without significant trauma or predisposing vascular risk factors is uncommon and may present a diagnostic challenge. In such cases, the clinical presentation can mimic inflammatory or demyelinating myelopathies, potentially leading to delayed diagnosis. A major hurdle in early diagnosis is the limited sensitivity of conventional MRI sequences during the hyperacute phase, which may remain normal for the first 24 to 48 hours. Recognition of characteristic magnetic resonance imaging (MRI) features, particularly longitudinally extensive T2 hyperintensity with anterior cord predominance, plays a crucial role in differentiating ischemic from non-ischemic causes. The role of diffusion-weighted imaging (DWI) is particularly vital in these instances, as its ability to detect restricted diffusion allows for the early identification of cytotoxic edema, providing objective evidence of infarction when standard sequences are inconclusive.

We report a case of SCI in a young patient following a seemingly trivial stretching maneuver, highlighting the diagnostic challenges, the potential contribution of embolic mechanisms like fibrocartilaginous embolism (FCE), and the importance of comprehensive MRI evaluation, including DWI, in establishing the diagnosis.

## Case presentation

A previously healthy 17-year-old male presented to the emergency department experiencing sudden bilateral upper limb weakness and numbness immediately after a routine stretching maneuver while studying. During the incident, the patient reported hearing a distinct "popping" sound. His neurological status deteriorated rapidly over the following hours, with weakness progressing to the lower limbs and resulting in a total inability to ambulate. While his immediate medical history was unremarkable, a later inquiry revealed he had sustained a fall four years prior for which he never sought medical attention, a detail that potentially correlated with findings on his cervical imaging.

Neurological examination upon arrival confirmed significant motor deficits, including distal weakness in the upper limbs, with handgrip strength of 3/5, and proximal lower-limb weakness, with hip flexion strength of 2/5 bilaterally. The patient exhibited classic dissociated sensory loss, characterized by reduced pain and temperature sensation below the C5 level, with absent deep tendon reflexes at both knees and ankles. These clinical features, particularly the preservation of dorsal column functions like proprioception, were highly suggestive of anterior spinal cord involvement.

**Figure 1:**
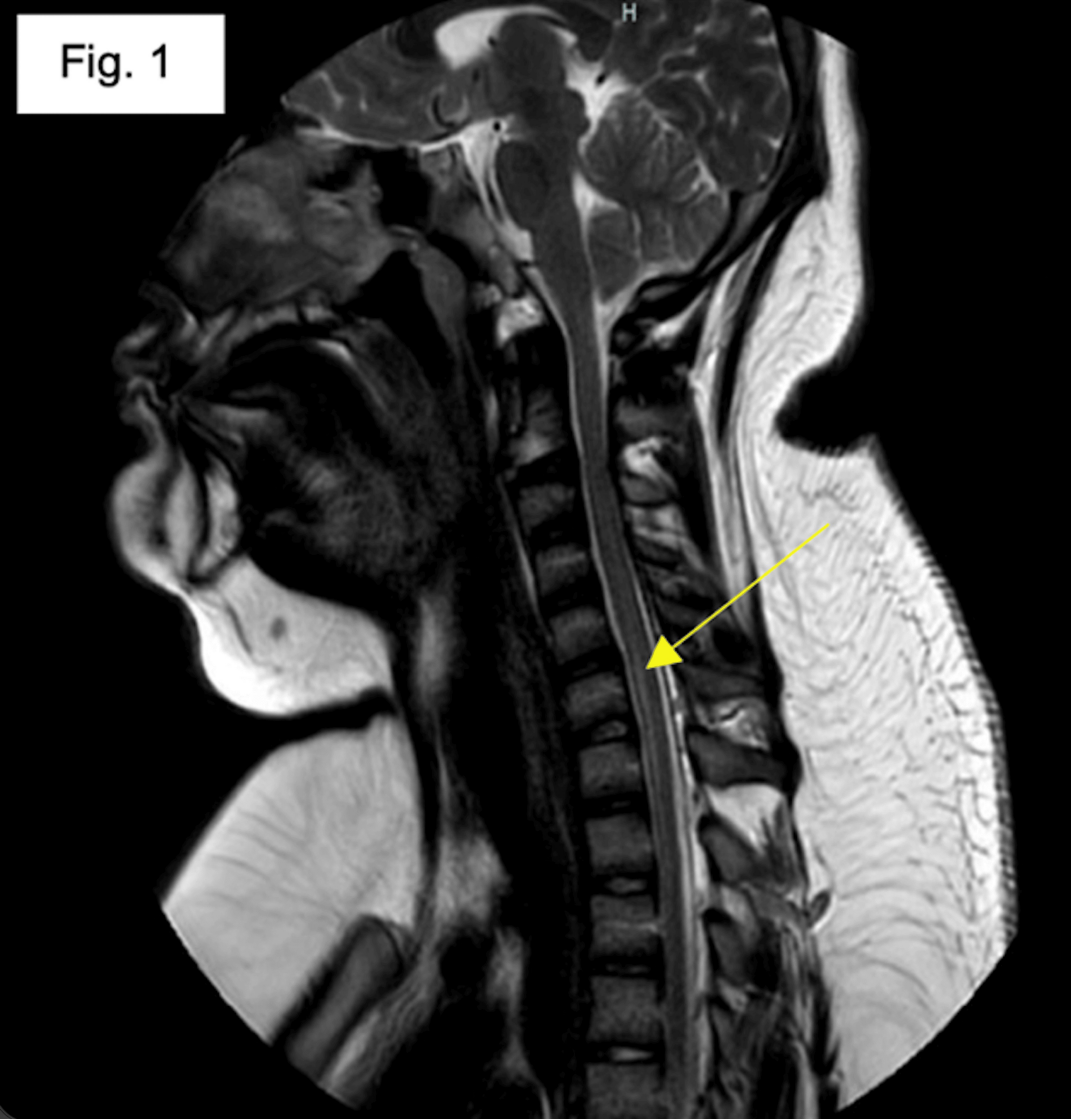
Sagittal T2-weighted cervical spine MRI showing intramedullary pencil-like hyperintensity extending from C5 to T3 (arrow).

**Figure 2:**
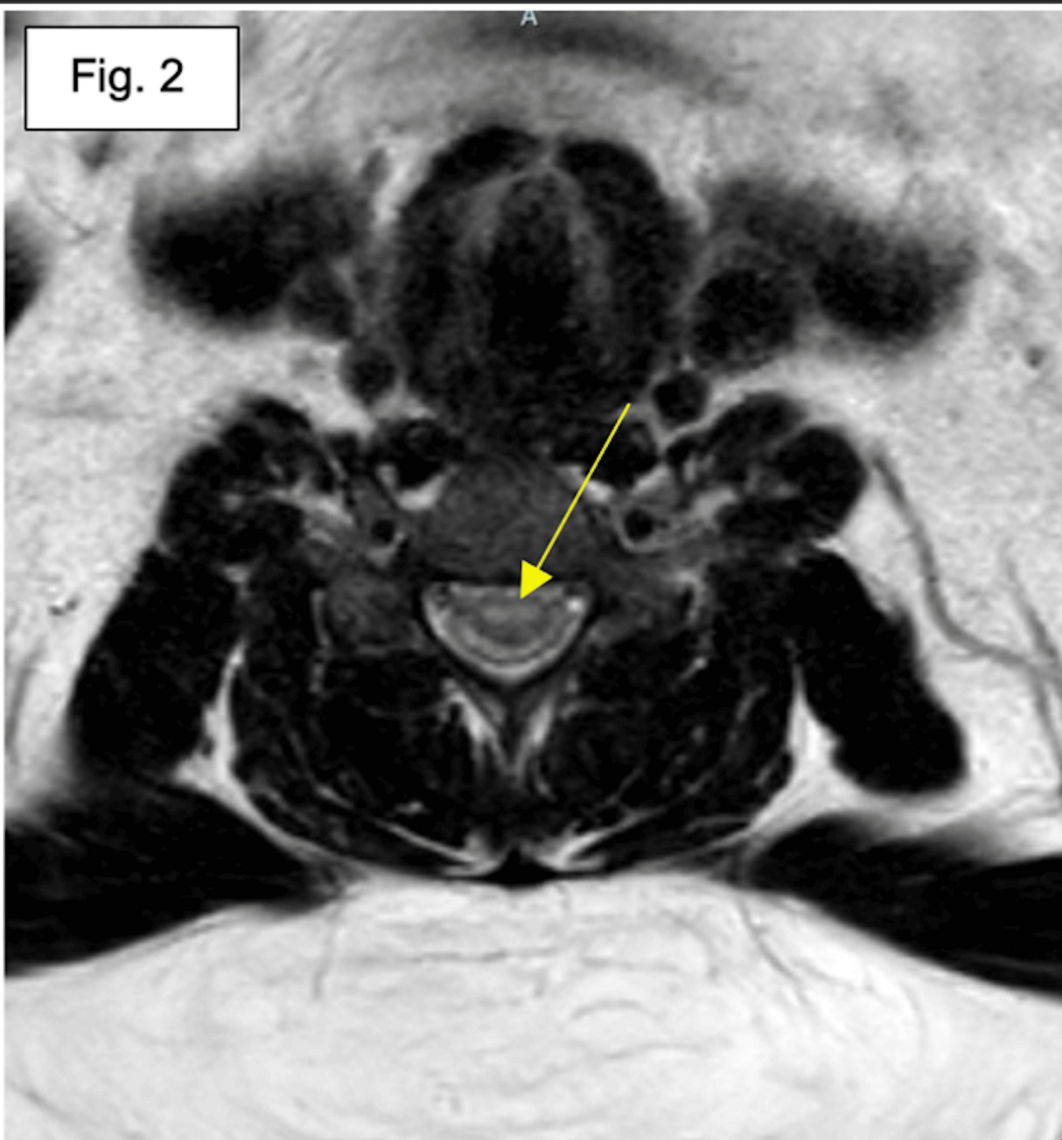
Axial T2-weighted cervical spine MRI at the C5 level demonstrating anterior column hyperintensity (arrow).

**Figure 3:**
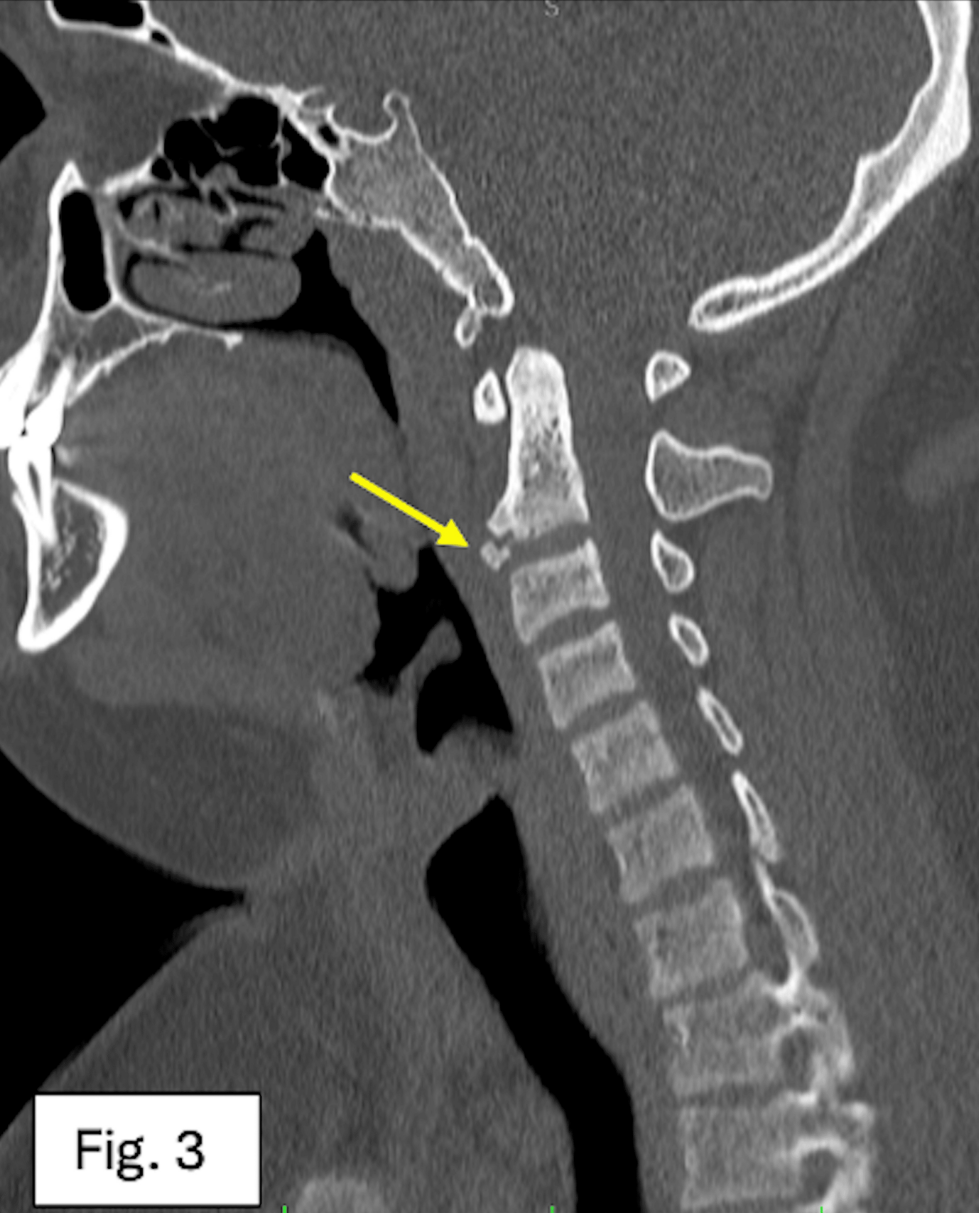
Sagittal CT cervical spine in bone window showing a small well-corticated bony fragment (arrow) at the anteroinferior aspect of the C2 vertebra.

Diagnostic investigations were prioritized to identify the cause of the acute quadriparesis. A cervical spine MRI performed approximately 24 hours after symptom onset demonstrated longitudinally extensive "pencil-like" T2-weighted intramedullary hyperintensities extending from C5 to T3 (Figure 1). These findings showed predominant anterior column involvement and a suspicious appearance of the "owl-eye sign" (Figure 2). Complementary imaging included a plain CT of the cervical spine, which identified a small, well-corticated bony fragment at the anteroinferior aspect of the C2 vertebra (Figure 3). CT angiography of the carotid and vertebral vessels showed opacified vertebral arteries, but the anterior spinal artery was not visualized, likely due to its small caliber and technical limitations.

The patient's clinical course was complicated by a febrile episode and leukocytosis during admission. Because these systemic signs can mimic an infectious or inflammatory process, an extensive workup was conducted, including blood cultures, an electrolyte panel, and thyroid function tests, all of which were unremarkable. Inflammatory markers, such as C-reactive protein and erythrocyte sedimentation rate (ESR), were normal, and screening for autoimmune markers, including aquaporin-4 receptor antibodies, was negative. Cerebrospinal fluid analysis from a lumbar puncture revealed no pleocytosis or abnormalities, effectively ruling out inflammatory and infectious myelopathies.

Given the overlap in early clinical and radiological features between vascular and inflammatory etiologies, an initial working diagnosis of transverse myelitis was made. Consequently, the patient was empirically started on a regimen of high-dose intravenous methylprednisolone, acyclovir, and broad-spectrum antibiotics to cover potential inflammatory and infectious causes during the hyperacute phase. However, following the negative results of the laboratory and cerebrospinal fluid (CSF) investigations and a reassessment of the abrupt symptom onset and mechanical trigger, the diagnosis was revised to anterior SCI. The final diagnosis favored ischemia over transverse myelitis due to the characteristic "pencil-like" MRI configuration and the lack of findings supporting a primary inflammatory process.

**Figure 4:**
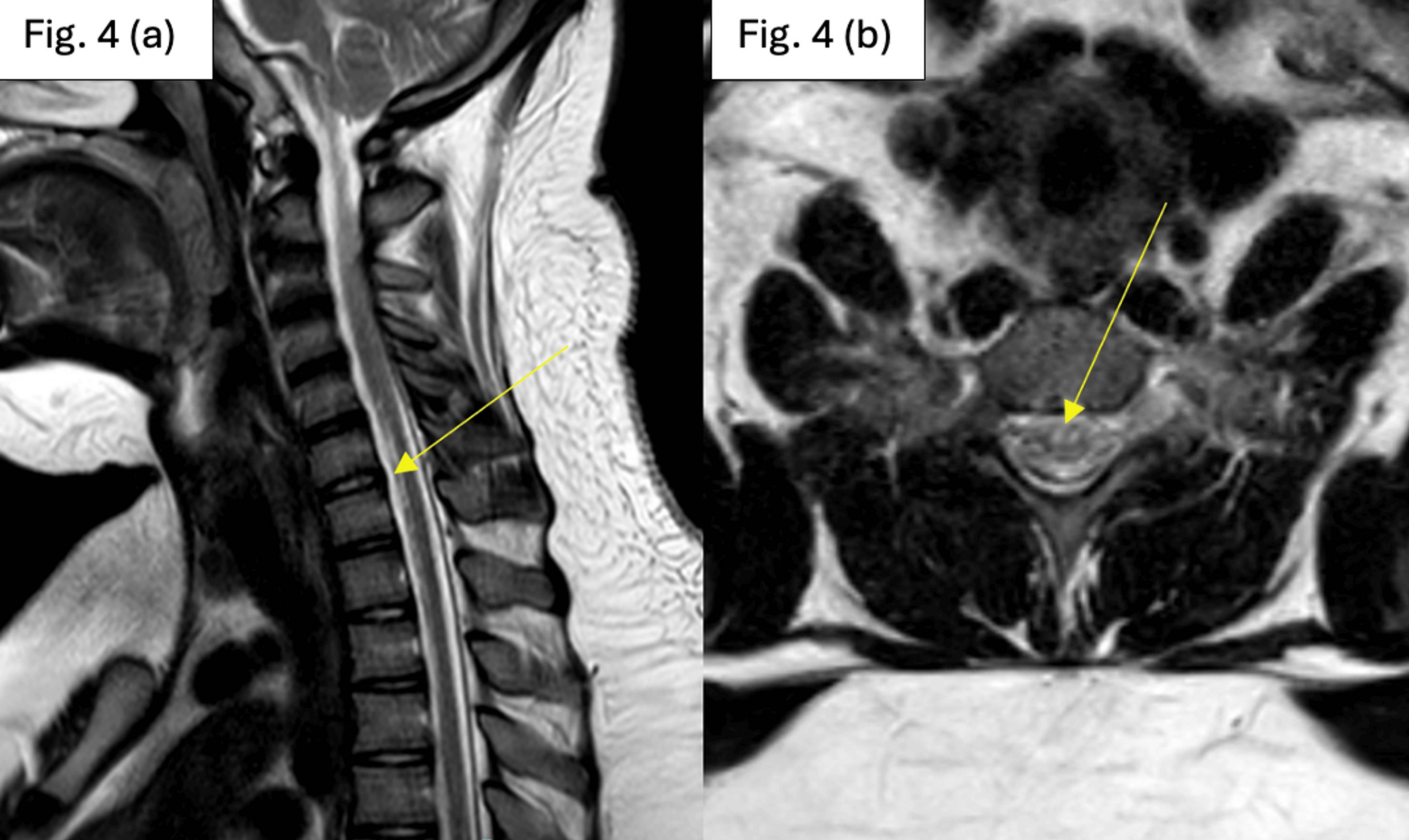
(a) A six-month follow-up sagittal T2-weighted cervical MRI shows high signal intensity from C5 to T3 level (arrow). (b) A six-month follow-up axial T2-weighted cervical MRI at C6 level showed owl’s eye appearance (arrow) of T2 hyperintensity.

Throughout his hospitalization, the patient showed gradual improvement in muscle strength. At a six-month follow-up in the neurology outpatient clinic, he demonstrated significant recovery with normalized muscle power in all four limbs, though residual weakness remained in both hands. A follow-up cervical MRI revealed persistent T2 hyperintensity involving the anterior horn from C5 to T3 (Figure 4a), now with a characteristic "owl’s eye" appearance from the C6 to T2 levels. These findings were interpreted as residual ischemic changes or evolving gliosis rather than permanent, established myelomalacia (Figure 4b).

## Discussion

SCI is a rare cause of acute myelopathy, accounting for approximately 1-2% of all strokes. Owing to its clinical overlap with inflammatory and infectious myelopathies, the diagnosis is often challenging, particularly in younger patients without traditional vascular risk factors [1,2].

The diagnosis in our patient was initially challenging because of the hyperacute neurological presentation accompanied by leukocytosis. Although leukocytosis may initially suggest an infectious process, it is a nonspecific finding that can also occur following acute ischemic injury as part of the systemic inflammatory response. Previous studies in acute ischemic stroke have demonstrated that elevated leukocyte counts are associated with greater stroke severity and poorer functional outcomes rather than indicating an underlying infection [3,4]. In our patient, the absence of fever, normal CSF findings, negative oligoclonal bands, and negative autoimmune and infectious investigations made inflammatory and infective myelopathies unlikely.

The hyperacute clinical presentation, together with the characteristic MRI findings, was crucial in establishing the diagnosis. SCI typically presents with sudden neurological deficits that reach maximal severity within hours, whereas inflammatory myelopathies generally progress over several days. Zalewski et al. proposed diagnostic criteria for spontaneous SCI that emphasize hyperacute symptom onset, exclusion of alternative diagnoses, and supportive MRI findings [5]. Our patient fulfilled these diagnostic criteria. MRI demonstrated longitudinal "pencil-like" T2 hyperintensity extending from C5 to T3 with predominant anterior cord involvement, consistent with infarction within the anterior spinal artery territory [1,2,5]. Follow-up MRI showed a more conspicuous "owl's eye" appearance, further supporting the diagnosis of anterior horn ischemia. Although DWI may improve diagnostic confidence in acute SCI, image quality remains limited by the small size of the spinal cord and susceptibility to physiological motion and magnetic susceptibility artifacts [6]. Nogueira et al. demonstrated that line-scan DWI reduces image distortion compared with conventional DWI and may improve the detection of SCI [7]. In our patient, the conventional DWI sequence was non-diagnostic because of severe image artifacts. The diagnosis was therefore established based on the characteristic hyperacute clinical presentation together with the evolution of MRI findings, including longitudinal anterior cord T2 hyperintensity giving the "owl's eye" appearance, fulfilling the proposed diagnostic criteria for spontaneous SCI [5].

CT demonstrated a small, well-corticated osseous fragment at the anteroinferior aspect of the C2 vertebral body. The absence of associated bone marrow edema on short tau inversion recovery (STIR) MRI favors a chronic rather than an acute osseous abnormality. This finding may represent sequelae of previous cervical trauma. However, there was no imaging evidence of acute cervical instability, vertebral artery injury, or direct vascular compromise to establish a causal relationship with the SCI.

The anterior spinal artery was not visualized on CT angiography. This finding should be interpreted with caution because visualization of the artery is often limited by its small caliber and the spatial resolution of CT angiography. Therefore, non-visualization alone should not be considered evidence of arterial occlusion [8].

The exact mechanism of SCI in our patient remains uncertain. FCE has been proposed as a rare cause of SCI following minor trauma or sudden physical exertion, particularly in young individuals [9]. The history of an abrupt stretching maneuver accompanied by an audible "pop" is consistent with this hypothesis. However, FCE remains a diagnosis of exclusion in living patients because definitive confirmation requires histopathological evidence, which is rarely available [9].

Previous reports have described SCI following seemingly trivial trauma, supporting the concept that clinically significant spinal cord ischemia may occur despite the absence of major spinal injury [9-11]. Although SCI is often associated with significant neurological disability, neurological recovery is variable and largely depends on the severity of the initial neurological deficit [12]. Our patient experienced substantial neurological improvement, with only mild residual hand weakness despite persistent T2 hyperintensity on follow-up MRI, which most likely represents chronic post-ischemic gliotic change rather than ongoing infarction.

This case highlights the importance of considering SCI in patients presenting with hyperacute myelopathy, even in the absence of traditional vascular risk factors. Correlation of the clinical presentation with characteristic MRI findings may facilitate an earlier diagnosis, avoid unnecessary investigations and treatment for inflammatory or infectious myelopathies, and support appropriate patient management.

## Conclusions

SCI should be considered in the differential diagnosis of young patients presenting with hyperacute myelopathy, even in the absence of traditional vascular risk factors or major trauma. Recognition of characteristic MRI findings, particularly longitudinal “pencil-like” T2 hyperintensity and anterior cord involvement, together with the clinical presentation, may facilitate earlier diagnosis and appropriate management. In our case, the diagnosis was supported by the clinical course and the evolution of MRI findings, although DWI was non-diagnostic due to imaging artifacts. The precise mechanism of infarction remains uncertain; while fibrocartilaginous embolism and minor mechanical trauma are possible explanations, a causal relationship could not be established. As a single case report, this study highlights an uncommon presentation of SCI and underscores the need for clinical awareness rather than establishing causality.
